# An Inquiry into the Degree of Certainty in Medicine, &c.

**Published:** 1849-01

**Authors:** 


### ARTICLE II.

*An Inquiry into the Degree of Certainty in Medicine, and into the Nature and Extent of its Powers, over Disease.* By ELISHA BARTLETT, M.D., Professor of the Theory and Practice of Medicine in Transylvania University, &c. Philadelphia: Lea & Blanchard, 1848. For sale by Keen & Brothers, Chicago.

The above is the title of a small volume filled with facts and arguments the most convincing and conclusive, to prove that medicine, as a science, has claims upon the the public confidence not to be disputed; and expressed in language so chaste and elegant that it may be classed with the very best literary productions of the day.

The author begins by stating, what we all know to be true that the enemies of the profession have been making unusual efforts of late to bring our honorable calling into disrepute, and to beget skepticism in the public mind as to its power of curing disease; and after commenting rather severely, but we think very justly, upon the views expressed by Dr. Forbes with regard to Homœpathy, in an article first published in the British and Foreign Medical Review, he informs his readers what the nature and character of the work is, in the following brief and concise language:

It seems to me high time, that a clear and earnest word should be spoken for the science which we study and teach, and for the art which we inculcate and practice. The interests of truth, of our profession, and of humanity, alike demand that the legitimate claims of medicine to the regard and confidence of mankind should be vindicated and maintained. And this is the task I have set myself. I wish to show as clearly and as positively as I can, the nature, and the degree of the certainty that belongs to medicine as a science, and as an art. In doing this, I shall deal but little in general assertions, unsustained by positive proofs, and not at all in empty and vague declamation; I shall state the reasons of the faith that I profess, and I shall exhibit the evidence upon which it rests.

It will be seen by the above quotation that the subject chosen is one well adapted to the pen of a writer like Dr. Bartlett, who is well known to be a good reasoner, capable of stating his propositions clearly, and expressing his views and feelings in a manner peculiarly attractive and interesting.

The work is evidently intended as a defence of the science of medicine, for the benefit of members of the profession, who, from want of enthusiasm, or a knowledge of modern improvements, have become lukewarm advocates and defenders of its merits. It is also well adapted to counteract the influence of the misrepresentation and abuse heaped upon the profession of late, by the selfish and unprincipled advocates of Homœpathy, Hydropathy, and the thousand other pathys which have recently sprung up to satisfy the morbid popular appetite for humbug and chicanery.

It is to be hoped that the book will be sought and read by all such as are willing to be influenced by well established facts rather than by mere assertions from such sources as these, and who are capable of distinguishing sound reasoning from sophistry based upon false assumptions.

Our author's closing remarks upon the medical delusions of the present day, as compared with our time-honored science, as it has existed, "embodied in the lives and labors of its professors for two thousand years," are made "in thoughts that speak, and words that burn," and are too good to be lost to our readers, even for so short a time as will be required for them to procure the work, we shall therefore quote them entire.

I have already remarked that my inquiry has had especial and exclusive reference to the science and art of medicine, as this science and art have been generally taught, understood, and practiced, for the last two thousand years. Before concluding my subject, it is proper enough that I should say a word or two about certain medical doctrines, and systems of practice, differing very widely from those which I have been examining and claiming to be sounder and more successful. I allude, as every body will at once understand, to homœopathy and hydropathy. It would have given me great pleasure to have instituted a fair and full comparison of these claims with those of what may be called, at least as a matter of convenience, and by way of marking the distinction, legitimate medicine. But the data essential to such a comparison do not exist; at any rate, I have been unable to procure them. I suppose the tables of Dr. Fleischmann, quoted by Dr. Forbes, furnished the most authentic and conclusive testimony that could then be found in favor of the superior excellence and efficacy of the homœopathic system. In regard to these tables, I can only repeat what I have already said, that they fail utterly and entirely of proving any such thing. Such tables prove nothing, except that a large proportion of certain diseases terminate in recovery, and a large proportion of others terminate in death,—a fact that stood in no particular need of any new proof. The nature of the evidence necessary in order to establish the actual and relative value of different remedies and methods of treatment, must be apparent enough to all who have gone through the present inquiry, although this subject has been only partially and incidentally considered. In the vast and complex science of medicine, this is the most difficult thing to be done. Its successful accomplishment requires a more thorough and exact knowledge of disease, a longer continued, more extensive, and more assiduous observation at its bed-side, sounder judgment, a more devoted loyality to truth, more entire freedom from all prejudice and passion, a nicer analysis, a more rigorous and inflexible logic, than are necessary to attain any other end in the science. Now, so far as I know, and I say this deliberately and without qualification, homœopathy has, in no single instance, on a scale of sufficient magnitude to be of any value, complied with the conditions which are absolutely necessary in order to ascertain the actual and comparative efficacy of its methods of treating disease. If it has done so, I have failed to find it out. If it can point to any such researches as those of Louis, and Jackson, and Grisolle, I am yet to learn whose and where they are.

I shall now lay down my pen and close this inquiry with a single reflection. I think that medical art as it has been embodied in the lives and labors of its professors, during two thousand years, has been worthy its high vocation, true to its great trust, faithful to its almost divine mission, and that this is more true of it now than ever it was before. I claim for it no exemption from the imperfections and frailties of all human concerns. I am very willing to admit that the personal conduct, and the scientific, professional, and general attainments of medical men have not always come fully up to the requirements and obligations of their position. Knaves find their way into all places, and

"Fools rush in where angels fear to tread."

Snobbishness, in the comprehensive meaning which Punch, in his genial pages of mingled wit and wisdom, has recently given to the term, is not confined to the other ranks and occupations of life; so much of it as appertains to the liberal professions has not been monopolized by the pulpit and the bar. Ignorance every day puts on the mask of knowledge, and pompous inanities pass current for the profoundest wisdom. Huge piles of stubble and rubbish are every year heaped up into shapeless ugliness, the fond builders believing all the while that they are rearing temples of adamant and marble, and the work goes bravely on to the admiring sound of braying asses, mistaken for the music of eternal fame. Sangrados ply the lancet and warm water in Paris as they did in Salamanca, and Sganarelles reason in the pages of the last journal as they do in those of Molière. Nevertheless, and notwithstanding all this, it is none the less true, that the obligations of the world to the science and the art of medicine, as they have been taught and practiced, are beyond all measurement or estimate. There is no process that can reckon up the amount of good which they have conferred upon the human race; there is no moral calculus that can grasp and comprehend the sum of their beneficent operations. Ever since the first faint dawn of civilization and learning, through

"The dark backward and abysm of time."

they have been the true and constant friends of the suffering sons and daughters of men. Through their ministers and disciples, they have cheered the desponding; they have lightened the load of human sorrow; they have dispelled or diminished the gloom of the sick chamber; they have plucked from the pillow of pain its thorns, and made the hard couch soft with the poppies of delicious rest; they have let in the light of joy upon dark and desolate dwellings; they have rekindled the lamp of hope in the bosom of despair; they have called back the radiance of the lustreless eye, and the bloom of the fading cheek; they have sent new vigor through the failing limbs; and finally, when exhausted in all their other resources, and baffled in their skill—handmaids of philosophy and religion—they have blunted the arrows of death, and rendered less rugged and precipitous the inevitable pathway to the tomb. In the circle of human duties, I do not know of any, short of heroic and perilous daring, or religious martyrdom and self-sacrifice, higher and nobler than those of the physician. His daily round of labor is crowded with beneficence, and his nightly sleep is broken that others may have better rest. His whole life is a blessed ministry of consolation and hope. Sweeter than the water-brooks to the panting hart are his kindly voice and his affectionate smile to the lonely presence of sickness, sorrow, and pain.

> "At his approach complaint grew mild,
> And when his hand unbarr'd the shutter,
> The clammy lips of fever smiled
> The welcome that they could not utter."

With these convictions of the powers and capabilities of our art, and of the general worthiness of its practitioners, we may rest assured, if we are only true to ourselves and to it, that the regard in which it has been held since the days of Hippocrates is in no danger of being permanently withdrawn. We must needs be visited occasionally by medical as by manifold other delusions; but it is a part of their nature always to pass rapidly away and to be soon forgotten. They are like fluttering eddies that cross the main current of the Mississippi or the Amazon; to him who happens to be caught in the tiny whirlpools, they may seem like the majestic tide of the great river itself, but they are soon inevitably lost and swallowed up in the rush of its resistless waters, and appear, to be seen no more. No, there is no danger. The work of two thousand years is not to be demolished by the noisy clamor of a few penny trumpets. As certainly as there is truth in the foregoing inquiry, will the present feeling of distrust towards our science and our art pass away. The ancient confidence will be restored; the old love will come back again, truer and deeper for the transient and passing estrangement. The constellations themselves—Orion and Pleiades—are sometimes apparently blotted out from the heavens, by the gorgeous glare of rockets and other artificial fireworks, kindled with sulphurous and nitrous compounds; but, courage! my friends, and a little patience,—the show will soon be over; the parti-colored flame that would rival and eclipse the planets is even now dying away; all that will remain of the blazing illumination will be some noisome gases in the atmosphere, and a few burnt out sticks on the ground; but lo! still looking down upon us, with their dear old smile of affectionate recognition, from their blue depths in the firmament, undimned in their brightness and unchangeable in their beauty, the everlasting stars.

